# Lactobacillus in autoimmune thyroid diseases: benefit or risk?

**DOI:** 10.3389/fimmu.2026.1905146

**Published:** 2026-08-03

**Authors:** Jin-min Yang, Peng-fei Hou, Shuai-ying Jia

**Affiliations:** 1 Thyroid-otolaryngology Department, Sichuan Clinical Research Center for Cancer, Sichuan Cancer Hospital & Institute, Sichuan Cancer Center, University of Electronic Science and Technology of China, Chengdu, China; 2 Department of Anesthesiology, Suining Municipal Hospital of Traditional Chinese Medicine (TCM), Sichuan, China; 3 Department of Anesthesiology, The Affiliated Hospital, Southwest Medical University, Luzhou, Sichuan, China; 4 Department of Pain Management, The Affiliated Hospital, Southwest Medical University, Luzhou, Sichuan, China

**Keywords:** autoimmune thyroid diseases, gut microbiota, immune homeostasis, lactobacillus, treatment strategy

## Abstract

Autoimmune thyroid diseases (AITDs), mainly Hashimoto’s thyroiditis and Graves’ disease, are common organ-specific autoimmune disorders driven by complex interactions among genetic susceptibility, immune dysregulation, environmental exposure, and microbial factors. Increasing evidence supports the relevance of the gut–thyroid axis, in which gut microbiota may influence thyroid hormone metabolism, intestinal barrier integrity, systemic inflammation, and thyroid-directed autoimmunity. Among gut commensals, Lactobacillus-related taxa have attracted attention because selected strains can modulate cytokine production, Treg/Th17 balance, short-chain fatty acid metabolism, epithelial barrier function, and inflammatory signaling pathways such as TLR4/NF-κB, AhR, and PPARδ-related pathways. However, most mechanistic evidence is derived from non-AITD animal, cellular, or metabolic disease models, and direct validation in AITD patients remains limited. This review summarizes current evidence on Lactobacillus-related strains in AITDs, emphasizing strain-specific effects, updated taxonomy, mechanistic plausibility, clinical heterogeneity, and translational limitations. We also discuss inconsistent microbial signatures, neutral or inconclusive clinical findings, and safety concerns, including probiotic-associated infection, antibiotic resistance genes, horizontal gene transfer, and potential pro-autoimmune effects. Overall, Lactobacillus-related interventions remain investigational, and future studies should prioritize standardized strain identification, disease-specific mechanisms, long-term safety, and well-designed clinical trials.

## 1 Introduction

Autoimmune thyroid diseases (AITDs), primarily including Graves’ disease (GD) and Hashimoto’s thyroiditis (HT), are the most common organ-specific autoimmune disorders. Epidemiological studies estimate a global prevalence of approximately 5–10%, with a markedly higher incidence in women, and a continuously rising trend in recent years. The pathogenesis of AITDs is highly complex and involves the interplay of genetic susceptibility, sex hormone levels, environmental factors (such as iodine intake and microbial infections), and immune dysregulation (1, 2). Central to their pathology is the breakdown of immune tolerance, leading to the production of thyroid-specific autoantibodies—such as thyrotropin receptor antibodies (TRAb), thyroid peroxidase antibodies (TPOAb), and thyroglobulin antibodies (TGAb)—which subsequently cause thyroid tissue damage and functional abnormalities (3, 4). Among the mechanisms of immune dysregulation, the imbalance between CD4^+^ T cell subsets, particularly T helper 17 (Th17) cells and regulatory T cells (Tregs), is considered to play a central role in the development of AITD. Th17 cells promote inflammatory responses through the secretion of proinflammatory cytokines such as IL-17 and IL-21, while Treg cells maintain peripheral immune tolerance via immunosuppressive cytokines, including IL-10 and TGF-β (5–8). Numerous studies have demonstrated that patients with AITDs exhibit elevated levels of Th17 cells and impaired Treg function in both peripheral blood and thyroid tissues, resulting in a disrupted Th17/Treg balance that contributes to persistent inflammation and disease progression (9–11).

In recent years, the gut microbiota has gained increasing recognition as a critical regulator of host immune homeostasis and has been increasingly implicated in the pathogenesis of AITDs. The concept of the “gut–thyroid axis” has been proposed, highlighting multidimensional interactions between gut microbes and the thyroid through immune, neuroendocrine, and metabolic pathways (12–14). However, mechanistic interpretations of gut microbiota–host interactions should be considered with caution, as microbial functions are highly strain-dependent and context-specific. Several studies have reported altered gut microbial composition in individuals with AITDs, characterized by a reduced abundance of bacteria belonging to genera such as Lactobacillus and Bifidobacterium, which contain strains that have been reported to exert beneficial effects in specific contexts, along with an increased relative abundance of opportunistic taxa including members of the Enterobacteriaceae family (15, 16). Such dysbiosis has been associated with impaired intestinal barrier integrity and altered microbial metabolite profiles, which may facilitate the translocation of bacterial components such as lipopolysaccharide (LPS) into the systemic circulation, thereby contributing to low-grade systemic inflammation. In parallel, reductions in short-chain fatty acid (SCFA)-producing bacteria may be linked to impaired differentiation and function of regulatory T cells, potentially affecting immune tolerance (17–19). Molecular mimicry between gut microbial antigens and thyroid autoantigens such as thyroid peroxidase and thyroglobulin has been proposed as a potential mechanism underlying microbial–immune cross-reactivity. This hypothesis has been suggested for certain bacterial species, including selected Lactobacillus and Bifidobacterium strains. However, current evidence remains limited and largely indirect, and strain-level validation is still lacking (20). Furthermore, the gut microbiota may indirectly influence thyroid function by modulating the bioavailability and absorption of trace elements such as iodine, selenium, zinc, and iron, which are essential for thyroid hormone synthesis and metabolism (21, 22). Collectively, these findings suggest that gut dysbiosis may contribute to the pathogenesis of AITDs through multiple pathways, and its characteristic alterations may hold diagnostic and prognostic value.

Among various microbiota-targeted interventions, lactic acid bacteria (LAB), a heterogeneous group comprising multiple genera and strains, have attracted considerable attention due to their established safety profiles, widespread use in food applications, and reported immunomodulatory potential (23). However, it should be emphasized that the biological effects of LAB are highly strain-specific and context-dependent, and cannot be generalized at the genus or group level. Evidence from experimental and clinical studies suggests that certain LAB strains may contribute to the maintenance of immune homeostasis by modulating Th17/Treg cell balance, enhancing intestinal barrier integrity, and influencing SCFAs production; however, these effects are not uniformly observed across all strains or study systems (24–27). In contrast, a limited number of studies have reported that specific LAB strains may, under particular genetic or environmental conditions, potentially trigger or exacerbate autoimmune responses through immune activation or putative molecular mimicry mechanisms. Nevertheless, these findings remain inconsistent and are largely based on strain-specific or model-dependent observations (28). Therefore, the role of LAB in the onset and regulation of AITDs remains incompletely understood. While certain LAB strains may represent promising immunomodulatory candidates, others may exhibit neutral or even adverse effects depending on host and environmental context. Elucidating strain-specific mechanisms of LAB in AITDs and rigorously evaluating their clinical safety and efficacy are therefore of significant theoretical and therapeutic importance.

## 2 Potential mechanistic pathways of lactobacillus strains relevant to autoimmune thyroid diseases

Lactic acid bacteria (LAB), a functional group of Gram-positive bacteria, include several genera such as Lactobacillus, Lactococcus, and Streptococcus. Within this group, Lactobacillus-related taxa represent a highly heterogeneous collection of species and strains with distinct genomic, metabolic, and immunomodulatory properties. Therefore, mechanistic interpretations should be considered at the strain level rather than generalized at the genus level. At present, direct experimental evidence demonstrating that specific Lactobacillus strains modulate thyroid-specific autoimmunity in patients with AITDs remains limited. Most mechanistic data are derived from non-AITD inflammatory, autoimmune, metabolic, epithelial barrier, or experimental animal models. Accordingly, the pathways discussed below should be interpreted as biologically plausible and hypothesis-generating, rather than as mechanisms already proven to operate in Hashimoto’s thyroiditis or Graves’ disease.

Specific Lactobacillus strains have been reported to regulate mucosal and systemic immunity through modulation of cytokine networks and T cell differentiation. For instance, *Lacticaseibacillus casei* and *Lactiplantibacillus plantarum* strains have been shown in experimental settings to modulate gut-associated lymphoid tissue by promoting anti-inflammatory cytokines, including IL-10 and TGF-β, while suppressing pro-inflammatory mediators such as IL-6 and TNF-α (29). Similarly, *Lacticaseibacillus rhamnosus GG* has been reported to influence CD4^+^ T cell differentiation and restore the Th17/Treg balance in non-AITD immune-mediated models (30). In AITDs, Th17/Treg imbalance and inflammatory cytokine dysregulation have been implicated in disease pathogenesis. Thus, these Lactobacillus-related effects provide a plausible immunological framework for future AITD studies. However, whether these strain-specific immunoregulatory effects occur in AITD patients has not been directly demonstrated.

Beyond cytokine modulation, certain Lactobacillus strains may exert immunometabolic effects through the production of bioactive lipid mediators. Several strains, including *Lactiplantibacillus plantarum*, *Furfurilactobacillus milii*, *Lentilactobacillus parabuchneri*, and *Lactiplantibacillus plantarum AKU 1009a*, have been reported to produce bioactive fatty acids and their derivatives. These metabolites, such as γ-linolenic acid and its downstream derivatives, including 13-hydroxy-γ-linolenic acid and 13-oxo-γ-linolenic acid, can activate peroxisome proliferator-activated receptor δ (PPARδ) more effectively than the parent compound (31–33). Because PPARδ signaling is involved in immune regulation, inflammation, and energy metabolism, Lactobacillus-derived lipid mediators may be relevant to endocrine–immune interactions. Nevertheless, this remains an indirect inference in the context of AITDs, and no current evidence demonstrates that Lactobacillus-derived PPARδ ligands regulate thyroid-specific autoimmunity in patients with Hashimoto’s thyroiditis or Graves’ disease.

Strain-specific effects have also been observed in the regulation of intestinal epithelial barrier function. For example, *Lactiplantibacillus plantarum HNU082* has been shown to enhance intestinal barrier integrity by upregulating tight junction proteins, including occludin and claudin-1 (34). In addition, *Lacticaseibacillus casei Zhang* participates in the fermentation of dietary fibers into SCFAs, including butyrate, acetate, and propionate (35). SCFAs can maintain intestinal pH, inhibit pathogenic bacterial growth, and act as signaling molecules involved in mucosal immune regulation and host metabolic communication. Impaired intestinal barrier function and altered microbial metabolites have been proposed as contributors to systemic immune dysregulation, and these processes may be relevant to the gut–thyroid axis. However, the contribution of Lactobacillus-mediated barrier protection or SCFA production to AITD pathogenesis has not been directly established.

**Figure 1:**
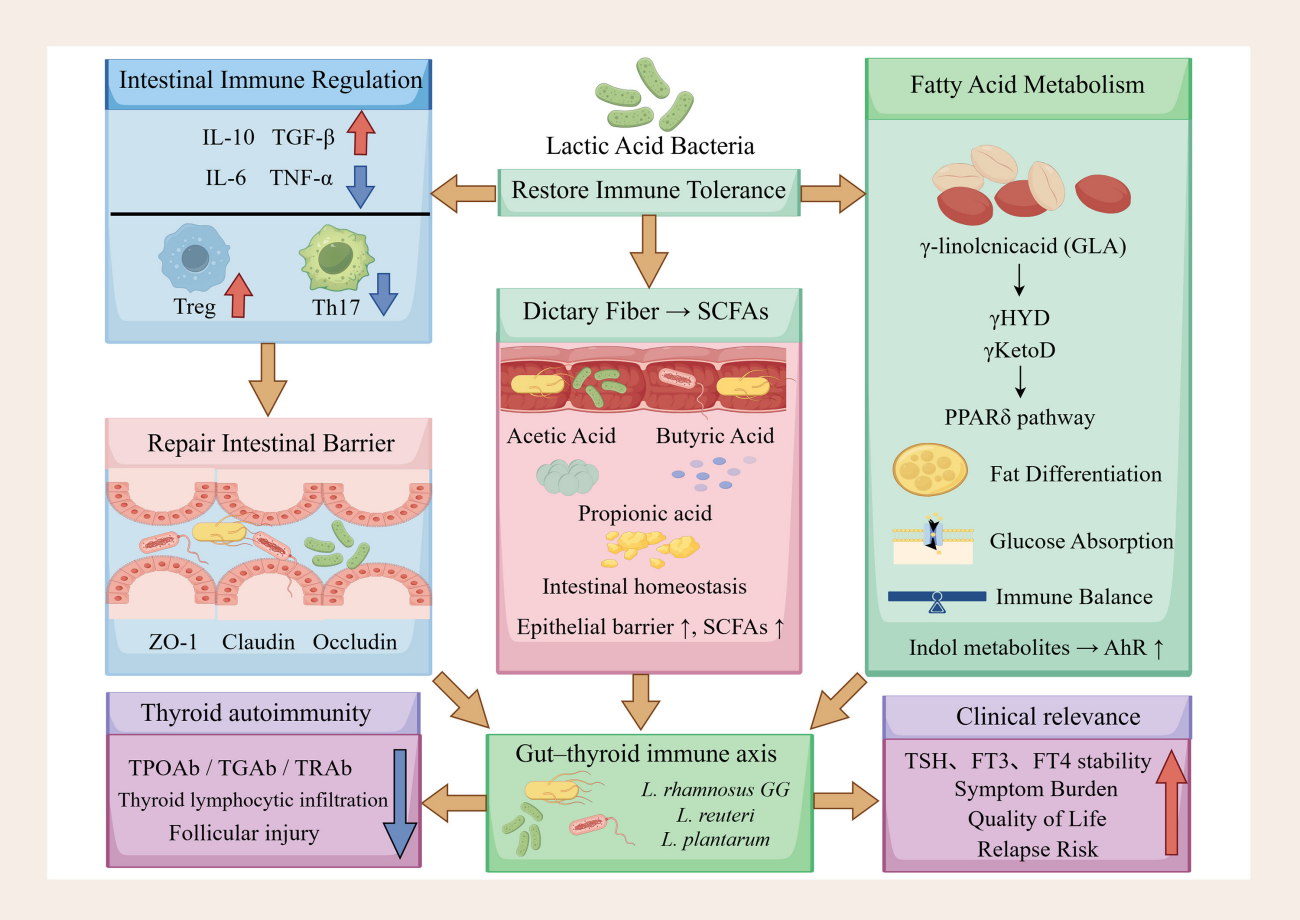
Lactic acid bacteria-mediated immunometabolic modulation in AITDs.

Other candidate pathways, including aryl hydrocarbon receptor (AhR) activation and suppression of inflammatory signaling pathways such as TLR4/NF-κB, should also be regarded as indirect and hypothesis-generating in AITDs. For example, *Lacticaseibacillus rhamnosus GG* has been characterized by its ability to induce IL-10 production and expand regulatory T cell populations in several experimental contexts (30, 36). *Limosilactobacillus reuteri* has been shown to metabolize aromatic amino acids into indole derivatives capable of activating the AhR, thereby contributing to the control of excessive immune activation (37, 38). In contrast, *Lactiplantibacillus plantarum* strains may contribute to immune regulation through enhancement of epithelial barrier integrity and production of SCFAs (39). Likewise, several Lactobacillus strains have been reported in non-AITD inflammatory models to attenuate TLR4/NF-κB signaling and reduce downstream inflammatory mediator production. These findings suggest possible mechanisms through which Lactobacillus-related taxa could influence systemic immune tone. However, direct evidence linking AhR activation or TLR4/NF-κB suppression by Lactobacillus strains to thyroid autoantibody production, thyroid lymphocytic infiltration, or clinical outcomes in AITD patients is currently lacking. A schematic summary of these pathways is provided in **Figure 1**.

Collectively, current evidence suggests that Lactobacillus-related strains may influence immune, metabolic, and epithelial barrier pathways that are biologically relevant to AITDs. However, the mechanistic evidence remains largely indirect. Until such evidence is available, Lactobacillus-related mechanisms in AITDs should be interpreted as hypothesis-generating rather than definitive.

## 3 Clinical and microecological evidence of lactobacillus strains in patients with AITDs

Recent studies have highlighted the pivotal role of specific Lactobacillus strains in modulating the gut microbiota and host immune responses, with growing attention to its potential clinical relevance in AITDs. Clinical microbiome analyses have reported associations between Lactobacillus-related taxa and thyroid function parameters. For instance, patients with thyroid nodules showed a significantly reduced relative abundance of Lactobacillus-assigned sequences compared with healthy controls (P < 0.001), which was positively correlated with the free triiodothyronine/free thyroxine (FT3/FT4) ratio (40). However, these data are derived from genus-level 16S rRNA profiling and do not allow resolution at the strain level, limiting mechanistic interpretation. Interventional studies using probiotic formulations have further suggested potential endocrine–microbiota interactions. Several clinical trials have demonstrated that supplementation with probiotics and prebiotics (including *Lacticaseibacillus casei*, *Lactobacillus acidophilus*, *Lacticaseibacillus rhamnosus*, *Lactobacillus delbrueckii subsp. bulgaricus*) can significantly reduce serum thyroid-stimulating hormone (TSH) levels in patients with subclinical hypothyroidism (mean difference: −0.19 mIU/L), improve the FT3/FT4 ratio, and alleviate related symptoms such as constipation (17, 41, 42). Nevertheless, these interventions typically involved mixed microbial formulations, and the specific contribution of individual Lactobacillus strains remains unresolved. Strain-resolved evidence is available for a limited number of well-characterized probiotics. In targeted interventional studies, *Lacticaseibacillus rhamnosus GG* has been shown to relieve symptoms associated with thyroid hormone withdrawal, including fatigue, constipation, and weight gain. These improvements were accompanied by significantly decreased levels of LPS in both serum and feces after treatment (43). Another study found that co-administration of *Bifidobacterium longum* with methimazole in Graves’ disease patients led to a marked reduction in TRAb levels—from 25.6 ± 5.2 IU/L to 1.2 ± 0.3 IU/L (44). However, this effect cannot be attributed to Lactobacillus specifically, as Bifidobacterium was the primary administered strain.

It is worth noting that the effects of Lactobacillus supplementation appear to be dose-dependent. In one study, fermented dairy products containing *Lacticaseibacillus paracasei strains (CNCM I-1518, CNCM I-3689)* and *Lacticaseibacillus rhamnosus CNCM I-3690* have been shown to enhance intestinal colonization of administered strains; however, no significant improvements in thyroid hormone markers were observed, suggesting that colonization alone may not be sufficient to induce endocrine effects (45). These findings suggest that the efficacy of Lactobacillus intervention may be influenced by strain specificity, dosage, treatment duration, and host factors, highlighting the need for individualized microbiome-based strategies.

Beyond interventional studies, microbial profiling analyses suggest that Lactobacillus-associated taxa may serve as potential biomarkers in AITDs. In Hashimoto’s thyroiditis, gut microbial signatures have been associated with disease status and thyroid hormone levels, suggesting potential diagnostic relevance (46). In GD, linear discriminant analysis (LDA) and LEfSe analysis have shown correlations between Lactobacillus-assigned abundance and TRAb levels, although these associations likely reflect broader microbial ecosystem shifts rather than strain-specific effects (47, 48). Moreover, alterations in genera such as Bacteroides, Alistipes, and Prevotella have been proposed as microbial markers to differentiate GD patients from healthy controls, achieving a classification accuracy of up to 85% (49).

Interestingly, the composition and abundance of Lactobacillus-related taxa are influenced by multiple host and environmental factors, including geographic region, dietary habits, sex, and age (50). Disease-specific alterations have also been reported; however, findings are inconsistent across cohorts. For example, A study in southwestern China found that GD patients had a higher abundance of Lactobacillus compared to healthy individuals (51). Another study analyzing45 GD patients and 59 healthy subjects reported reduced α-diversity of gut microbiota in the GD group, with significant depletion of Bifidobacterium, Holdemanella, and Anaerostipes, alongside increased levels of Bacteroides and Lactobacillus (47). In a separate cohort of 20 AITD patients and 30 healthy controls, the Firmicutes/Bacteroidetes ratio was significantly decreased in the patient group (P < 0.001), with concurrent reductions in beneficial genera such as Lactobacillus and Bifidobacterium (52). These discrepancies further highlight the context-dependent and non-uniform nature of Lactobacillus-associated changes. The gut microbiota alterations and clinical correlations in AITDs are summarized in **Table 1**. Overall, current evidence suggests that Lactobacillus-related taxa may be associated with immune–endocrine modulation and may serve as potential diagnostic biomarkers in AITDs. However, due to the strong strain specificity of probiotic effects, along with methodological limitations in taxonomic resolution, current findings should be interpreted cautiously. Future studies employing strain-level metagenomics and controlled mono-strain interventions are required to clarify causal relationships and therapeutic potential.

**Table 1:** Summary of gut microbiota alterations and clinical correlations in AITDs.

Disease type	Gut microbiota alterations	Lactobacillus trend	Thyroid-related indicators	Reference
Hashimoto’s Thyroiditis	↑: Blautia,Roseburia, Ruminococcus_torques_group, Romboutsia, Dorea, Fusicatenibacter, Eubacterium_hallii_group; ↓: Fecalibacterium, Bacteroides, Prevotella_9, Lachnoclostridium	Not reported	7 clinical parameters correlated with microbiota, Biomarkers showed AUC = 0.91 (exploration), AUC = 0.88 (validation)	(46)
Graves’ Disease	↓: Blautia, [Eubacterium]_hallii_group, Anaerostipes, Collinsella, Dorea, unclassified_f_Peptostreptococcaceae, [Ruminococcus]_torques_group, Firmicutes; ↑: Bacteroides, Lactobacillus, Bacteroidetes	Increased in GD patients; subgroup analysis suggests Lactobacillus may play a key role in autoimmune thyroid disease pathogenesis.	Nine genera showed significant correlations with thyroid function tests (specific tests not named). Blautia implicated in metabolic pathways during hyperthyroid state. Diagnostic model using top 9 genera: AUC = 0.8109 (CI: 0.7274–0.8945)	(47)
Graves’ Disease	↑ in GD: Lactobacillus, Veillonella, Streptococcus; After treatment ↓: Blautia, Corynebacter, Ruminococcus, Streptococcus; After treatment ↑: Phascolarctobacterium	Significantly increased in untreated GD patients; shows positive correlation with TRAb level; considered a potential biomarker of GD pathogenesis.	TRAb positively correlated with Lactobacillus and Ruminococcus; TRAb, TPOAb, TGAb all negatively correlated with Synergistetes (R < -0.6, P < 0.05); Phascolarctobacterium negatively correlated with TRAb, increased after recovery.	(48)
Graves’ Disease	Decrease in SCFA-producing bacteriaand SCFAs (especially propionic acid); Bacteroides, Alistipes, and Prevotella distinguish GD with 85% accuracy; B. fragilis (YCH46 strain) restores Treg/Th17 balance via propionic acid	Not reported	Not directly reported; thyroid dysfunction inferred from increased GD incidence in mouse model	(49)
Graves’ Disease	Increased abundance of Firmicutes, Proteobacteria, Actinobacillus; Higher Firmicutes/Bacteroidetes ratio; Genera Oribacterium, Mogibacterium, Lactobacillus, Aggregatibacter significantly higher in GD group	↑ Increased in GD group	Not directly measured; study focuses on microbiota differences without specific thyroid hormone data	(51)
AITDs, including Hashimoto’s Thyroiditis and Graves’ Disease	Firmicutes/Bacteroidetes ratio significantly ↓ in ATD patients (P <.001); ↓ Abundance of beneficial bacteria: A. mucinophilia, Bifidobacterium, Lactobacillus,F. prausnitzii;	Decreased in AITD group	TRAb (GD) positively correlated with Bacteroidetes (P = .001); Anti-TPO (HT) positively correlated with Bacteroidetes (P = .018)	(52)

## 4 Evidence from AITD and non-AITD models: mechanistic plausibility and translational limitations

In recent years, increasing attention has been directed toward the potential role of Lactobacillus-related strains in thyroid autoimmunity. However, the available preclinical evidence is heterogeneous. Only a limited number of studies have directly examined Lactobacillus strains in animal models of AITDs, whereas a substantial proportion of mechanistic evidence comes from non-thyroid inflammatory, metabolic, neurological, fish, or zebrafish models. These studies provide biological plausibility for immune, metabolic, epithelial barrier, and microbiota-mediated mechanisms, but they should not be interpreted as direct evidence for AITD pathogenesis or treatment.

Several Lactobacillus strains, including *Lacticaseibacillus rhamnosus GG*, *Limosilactobacillus reuteri*, and *Lacticaseibacillus casei*, have been reported in non-AITD inflammatory or metabolic models to downregulate pro-inflammatory cytokines, including interleukin-6 (IL-6) and tumor necrosis factor-alpha (TNF-α), enhance intestinal barrier integrity, and restore gut microbial homeostasis (53–55). These findings suggest that Lactobacillus strains may reshape the intestinal microecosystem and modulate systemic immune-inflammatory responses. Because cytokine dysregulation, intestinal barrier impairment, and gut microbiota alterations have been implicated in thyroid autoimmunity, these mechanisms may be relevant to the gut–thyroid axis. Nevertheless, their relevance to AITDs remains indirect, as immune responses in colitis, obesity, Parkinson’s disease, or other non-thyroid disease models are not driven by thyroid-specific autoantigens and do not fully reproduce the endocrine and autoimmune features of Hashimoto’s thyroiditis or Graves’ disease. Moreover, Certain Lactobacillus strains may also influence trace element metabolism, particularly selenium metabolism. Some strains can promote the conversion of inorganic selenium into organic forms, such as selenomethionine and selenocysteine, thereby improving selenium bioavailability and potentially enhancing antioxidant capacity and immune regulation (56). Given the proposed relationship between selenium status, oxidative stress, and thyroid autoimmunity, this pathway may be relevant to AITDs. However, current evidence does not yet establish that Lactobacillus-mediated selenium metabolism directly improves thyroid autoantibody profiles, thyroid function, or clinical outcomes in patients with Hashimoto’s thyroiditis or Graves’ disease.

Some studies suggest that Lactobacillus-related interventions may influence thyroid hormone homeostasis, but these findings should also be interpreted cautiously. For example, *Lacticaseibacillus rhamnosus* administration increased serum concentrations of T3 and T4 by approximately 20% and 15%, respectively, in juvenile tilapia (57). Although this observation indicates that Lactobacillus may affect thyroid hormone-related physiology in aquatic species, its direct relevance to human AITDs is limited because fish thyroid regulation differs substantially from mammalian thyroid autoimmunity. Similarly, transplantation of gut microbiota from hypothyroid patients into mice led to a 10%–15% reduction in FT4 (58), supporting a possible microbiota–thyroid hormone connection. However, this model reflects altered thyroid hormone metabolism rather than Lactobacillus-specific modulation of thyroid autoimmunity. More disease-relevant evidence comes from experimental models of Graves’ disease and Hashimoto’s thyroiditis. In a Graves’ disease model, supplementation with *Lacticaseibacillus rhamnosus* suppressed the elevation of T4 and reduced lymphocytic infiltration in thyroid tissue (59). Safety evaluations have further shown that strains such as *Lacticaseibacillus rhamnosus HN001* and *Bifidobacterium animalis subsp. lactis HN019* did not induce HT in animal models, indicating a favorable safety profile for potential therapeutic application (56).

In an experimental Hashimoto’s thyroiditis model, Lactobacillus intervention was reported to attenuate chronic thyroid inflammation, preserve thyroid follicular structure, and reduce thyroid autoantibodies, including TPOAb. Mechanistically, these effects were associated with suppression of the TLR4/MyD88/NF-κB signaling pathway and reduced expression of inflammatory mediators such as IL-1β and TNF-α (11). Compared with evidence from non-thyroid disease models, these findings provide more direct support for a potential role of Lactobacillus strains in thyroid-specific autoimmune inflammation. However, they remain preclinical and require validation in human AITD cohorts. Additionally, Microbial metabolites produced by certain Lactobacillus strains, such as indole-3-lactic acid and indole-3-propionic acid, can activate the aryl hydrocarbon receptor pathway and may enhance regulatory T cell function in experimental settings (60, 61). These mechanisms are biologically relevant because immune tolerance and Treg dysfunction are involved in autoimmune diseases, including AITDs. Nevertheless, most evidence for Lactobacillus-mediated AhR activation comes from non-AITD models. Therefore, AhR-related immune regulation should be regarded as a plausible but unproven mechanism in thyroid autoimmunity. Overall, evidence from non-thyroid disease models, fish, zebrafish, and microbiota-transfer experiments supports the broader concept that Lactobacillus-related strains can influence immune regulation, epithelial barrier function, microbial metabolites, trace element metabolism, and thyroid hormone-related physiology. However, these models differ from AITDs in target-organ specificity, thyroid autoantigen involvement, endocrine feedback regulation, and disease-specific immune microenvironments. Therefore, such findings should be used to generate hypotheses rather than to establish causality in AITDs. Future studies should prioritize AITD-specific animal models and well-designed clinical studies assessing thyroid autoantibodies, thyroid hormone profiles, inflammatory markers, gut microbiota composition, and clinical outcomes after strain-specific Lactobacillus interventions.

## 5 Clinical evidence for lactobacillus-containing probiotic formulations in AITDs: positive signals, neutral findings, and limitations

In recent years, probiotic formulations, often containing Lactobacillus-related strains, have been investigated as potential adjunctive interventions in thyroid-related disorders, including Graves’ disease, Hashimoto’s thyroiditis, gestational hypothyroidism, subclinical hypothyroidism, and thyroid cancer patients undergoing thyroid hormone withdrawal. However, the current clinical evidence remains preliminary and heterogeneous. Most available studies are limited by small sample sizes, short intervention periods, multi-strain formulations, variable background treatments, and inconsistent clinical endpoints. Therefore, the available data should be interpreted as exploratory clinical signals rather than evidence supporting routine Lactobacillus-based therapy in autoimmune thyroid diseases.

Some trials have reported favorable changes in thyroid-related outcomes. In patients with hypothyroidism, a randomized controlled trial reported that a probiotic formulation containing Lactobacillus and Bifidobacterium was associated with a modest reduction in serum TSH levels, with a mean difference of −0.19 mIU/L, and fewer levothyroxine dose adjustments in the intervention group than in the control group (62). However, the magnitude of TSH reduction was small, and the study did not establish whether the observed effect was attributable to a specific Lactobacillus strain, Bifidobacterium component, or the combined formulation.

In the context of GD, one randomized controlled trial reported that a probiotic intervention combining *Bifidobacterium longum* with methimazole led to a significant reduction in TRAb levels, from 25.6 ± 5.2 IU/L to 1.2 ± 0.3 IU/L, outperforming the monotherapy group (P < 0.05) (44). Although this finding suggests that microbiota-targeted adjunctive therapy may influence autoimmune activity, the formulation was not Lactobacillus-specific. Therefore, this study should not be interpreted as direct evidence for Lactobacillus efficacy in Graves’ disease.

Among pregnant women with hypothyroidism, interventions combining probiotics and prebiotics have also shown clinical benefits. After 21 days of supplementation, significant decreases in TSH levels were observed (*P* < 0.05), along with improvements in inflammatory markers (IL-6, TNF-α) and lipid profiles (total cholesterol and low-density lipoprotein). Notably, the probiotic formulation used in this study contained *Lactobacillus acidophilus*. This suggests that interventions including *Lactobacillus acidophilus* may contribute to the regulation of thyroid-related metabolism and inflammation. However, the specific contribution of this strain still needs to be confirmed in controlled studies (63). Furthermore, in thyroid cancer patients undergoing hormone withdrawal, probiotic administration alleviated nonspecific symptoms such as fatigue, constipation, and weight gain. The probiotic formulation included *Lactobacillus acidophilus*, and these effects were accompanied by reductions in lipopolysaccharide levels in both serum and feces, supporting the potential role of Lactobacillus-containing probiotic interventions in maintaining intestinal barrier integrity and mitigating systemic inflammation (43).

However, clinical responses remain heterogeneous. While some patients respond well to probiotic interventions, others show little to no improvement. This variability may be attributed to differences in gut microbiota composition, host immune-genetic background, the presence of comorbid conditions, intervention duration, and the strain composition of probiotic formulations. In subclinical hypothyroidism, supplementation with a multi-strain probiotic formulation containing *Lacticaseibacillus casei*, *Lactobacillus acidophilus*, *Lacticaseibacillus rhamnosus*, and *Lactobacillus delbrueckii subsp. bulgaricus*, combined with prebiotics, was associated with reductions in TSH levels (MD = −0.19 mIU/L) and improvements in the FT3/FT4 ratio. These findings provide additional evidence that specific Lactobacillus-containing probiotic combinations may influence thyroid hormone regulation (41). **Table 2** presents a summary of the randomized controlled trial on synbiotic supplementation. Overall, current clinical evidence suggests that selected probiotic formulations may influence thyroid-related biochemical indices, inflammatory markers, gastrointestinal symptoms, or quality-of-life parameters in some settings. However, the evidence remains insufficient to support Lactobacillus therapy as a routine adjunctive treatment for AITDs. Future trials should include larger sample sizes, longer follow-up, standardized strain identification, defined dosing regimens, appropriate placebo controls, and AITD-specific outcomes, including TPOAb, TgAb, TRAb, thyroid hormone stability, levothyroxine or antithyroid drug requirements, relapse rates, and adverse events.

**Table 2:** Summary table of a randomized controlled trial on synbiotic supplementation in hypothyroid patients.

Disease type	Intervention	Clinical outcomes	Possible mechanism	Reference
primary hypothyroidism	VSL#3^®^ (high-potency Lactobacilli+ Bifidobacteria) combined with LT4	No significant differences in TSH, fT3, fT4 levels; FT3/fT4 ratio less correlated to TSH in probiotics group (1st visit); Fewer LT4 dose adjustments in probiotics group (p = 0.007)	Probiotics may modulate gut microbiota and help stabilize thyroid hormone levels, reducing fluctuations	(62)
clinical hypothyroidism	Probiotics + prebiotics combined with LT4	Higher SIBO prevalence in hypothyroid pregnant women (48.5% vs 24.8%); Methane-positive rate and gas levels reduced post-treatment; Constipation and bloating improved; TSH, hsCRP, IL-6, TNF-α, TC, LDL levels decreased	Regulation of gut microbiota may reduce SIBO burden, inflammation, and improve thyroid/lipid metabolism	(63)
Hypothyroidism	500 mg/day synbiotic supplement for 8 weeks	Decreased TSH; Reduced levothyroxine dosage; Lower fatigue severity score; Increased FT3 (within-group only); CRP increased (not significant between groups); No changes in TPOAb or blood pressure	Gut microbiota modulation may enhance levothyroxine absorption and thyroid hormone activity, reducing TSH and fatigue	(41)

## 6 Controversies and challenges in the clinical application of lactobacillus for AITDs

### 6.1 Potential pro-autoimmune and pathogenic pathways of lactobacillus-related strains

Although many Lactobacillus-related strains have been investigated for their immunoregulatory properties, their effects are not uniformly anti-inflammatory. Depending on the strain, host immune status, disease context, dosage, and background microbiota, Lactobacillus-related taxa may also exert proinflammatory or immune-activating effects. This possibility is particularly relevant for autoimmune diseases, in which excessive or misdirected immune activation may aggravate disease activity rather than restore immune tolerance.

One potential concern is molecular mimicry. Bacterial antigens may share structural similarities with host proteins and thereby promote cross-reactive immune responses in genetically susceptible individuals. Although direct evidence linking Lactobacillus-derived antigens to thyroid-specific molecular mimicry remains limited, this mechanism is biologically plausible and has been proposed in other autoimmune contexts (20). In AITDs, where immune responses against thyroid peroxidase, thyroglobulin, or the TSH receptor are central to disease pathogenesis, future studies should determine whether specific bacterial antigens can induce or amplify thyroid-directed autoreactivity.

In addition, some Lactobacillus-related strains may enhance proinflammatory immune responses under certain conditions. Experimental studies in central nervous system autoimmunity have shown that specific Lactobacillus strains can aggravate autoimmune inflammation rather than suppress it (26, 27). These findings indicate that Lactobacillus-related organisms are not universally protective and may promote pathogenic immune responses in selected disease settings. Although CNS autoimmunity differs from thyroid autoimmunity in target organ, antigen specificity, and tissue microenvironment, these studies provide an important cautionary example that strain-specific immune activation may worsen autoimmune pathology.

Potential mechanisms by which Lactobacillus-related strains may exacerbate autoimmunity include activation of antigen-presenting cells, increased production of proinflammatory cytokines, promotion of Th1 or Th17 responses, alteration of Treg/Th17 balance, disruption of microbial community structure, or excessive stimulation of pattern-recognition receptor pathways. Such effects may be more likely in hosts with active inflammation, impaired intestinal barrier function, dysbiosis, genetic susceptibility, or concurrent immune-modulating therapy (64–67). Therefore, the same strain may have different immunological consequences depending on the host and disease context.

For AITDs, direct evidence that Lactobacillus-related strains exacerbate thyroid autoimmunity is currently insufficient. However, given the central role of thyroid-specific autoantibodies, lymphocytic thyroid infiltration, and dysregulated T cell responses in Hashimoto’s thyroiditis and Graves’ disease, potential pro-autoimmune effects should not be overlooked. Future studies should evaluate not only beneficial outcomes but also possible worsening of thyroid autoantibody titers, thyroid hormone instability, inflammatory markers, symptom burden, and disease relapse. These concerns reinforce the need for strain-specific, disease-specific, and safety-oriented evaluation before Lactobacillus-related interventions are considered for clinical use in AITDs.

### 6.2 Individual variability: a key limitation in clinical application

Significant individual differences in response to probiotic interventions containing Lactobacillus strains represent a major challenge in the clinical application of microbiota-based therapies for AITDs. Core determinants of such variability include baseline gut microbiota composition, genetic susceptibility, coexisting conditions, and differences in treatment regimens. In addition to host-related factors, strain-specific characteristics, including adhesion capacity, metabolite production, and immunomodulatory properties, may substantially influence clinical outcomes. For example, a randomized controlled trial reported that while some hypothyroid patients showed notable reductions in TSH following probiotic treatment, others did not benefit. This disparity was closely related to the initial abundance of Bifidobacterium species (62). Similarly, in patients with Graves’ disease, the degree of TRAb reduction after treatment with *Bifidobacterium longum* was associated with host IL-10 gene polymorphisms (44), suggesting that immune-genetic background may influence the immunomodulatory effects of specific probiotic strains.

The presence of comorbidities also plays a critical role in treatment outcomes. In patients with autoimmune liver disease coexisting with AITD, probiotic therapy resulted in less improvement in thyroid function compared to those with AITD alone (68). In contrast, pregnant women with hypothyroidism exhibited better responses to probiotic intervention, possibly due to immune and hormonal modulation during pregnancy (63). The composition of probiotic regimens—including strain selection, dosage, and duration—also significantly affects efficacy. One study found that increased consumption of a Lactobacillus-containing fermented yogurt (specifically containing *Lacticaseibacillus paracasei CNCM I-1518*, *Lacticaseibacillus paracasei CNCM I-3689*, and *Lacticaseibacillus rhamnosus CNCM I-3690*) was associated with greater improvement in thyroid function compared with lower intake; however, the specific contribution of individual bacterial strains remains unclear (45). These findings underscore the highly individualized nature of probiotic therapy in AITDs and highlight the need for precision-medicine approaches to identify potentially effective strains and optimize treatment parameters.

### 6.3 Long-term safety: risk assessment in vulnerable populations

Long-term safety remains a critical consideration in the clinical use of Lactobacillus-containing probiotic formulations, as safety profiles may vary considerably among individual strains, formulations, doses, and host conditions. Potential safety concerns associated with probiotic supplementation include alterations in gut microbial ecology, rare systemic infections, metabolic effects, and the potential carriage or transfer of antibiotic resistance genes. Therefore, safety should not be assumed to be uniform across Lactobacillus-related taxa. Available evidence suggests that some well-characterized strains have favorable long-term safety profiles. A 3-year follow-up study found that infants fed formula supplemented with *Limosilactobacillus fermentum CECT5716* maintained a stable gut microbiota composition and showed no significant differences in growth and development compared with controls, indicating good long-term tolerance of this specific strain (69). Subchronic toxicity studies also demonstrated that even at high doses, up to 92 × 10^8^ CFU/kg/day, *Lacticaseibacillus rhamnosus GG* did not induce adverse effects in animal models (70). These findings support the safety of *Lactobacillus fermentum CECT5716* and *Lacticaseibacillus rhamnosus GG* specifically, but cannot necessarily be generalized to all Lactobacillus strains.

However, Certain populations require particular caution, including immunocompromised individuals, critically ill patients, patients with central venous catheters, those with impaired intestinal barrier integrity, recent gastrointestinal surgery, severe comorbidities, or antibiotic-induced dysbiosis. In the context of autoimmune thyroid diseases, additional attention may be needed for patients receiving antithyroid drugs, because rare but serious adverse events such as agranulocytosis may increase susceptibility to infection. In these vulnerable populations, prolonged administration of live Lactobacillus-containing products may theoretically increase the risk of bacteremia or opportunistic infection, although the reported incidence remains very low (71). Probiotic-associated invasive infections are uncommon but clinically relevant. Case reports and reviews have described Lactobacillus-related bacteremia, sepsis, and endocarditis, particularly in patients with severe underlying diseases, immune suppression, intensive care admission, central venous catheters, or disrupted mucosal barriers (72–74). Potential mechanisms include bacterial translocation across a damaged intestinal barrier and contamination or colonization of intravascular devices. Therefore, although the absolute risk appears low, probiotic-associated bloodstream infection should be considered an important translational safety concern when live bacterial products are used in vulnerable populations. Another unresolved safety issue is the potential carriage and transfer of antibiotic resistance genes. Some Lactobacillus-related strains possess intrinsic resistance phenotypes that may be non-transferable, whereas acquired resistance genes located on plasmids, transposons, or other mobile genetic elements may theoretically be transferred horizontally to commensal or pathogenic bacteria (75, 76). Although *in vivo* evidence for clinically meaningful horizontal gene transfer from probiotic strains remains limited, this possibility is particularly important in patients exposed to antibiotics or in healthcare environments where antimicrobial resistance pressure is high. Accordingly, future safety assessments should distinguish intrinsic resistance from acquired, mobile resistance determinants.

Additionally, Extended probiotic use may also alter gut microbial ecology and metabolite profiles. For example, increased production of short-chain fatty acids may influence glucose and lipid metabolic homeostasis (58). Safety profiles also vary across strains. For instance, *Lactiplantibacillus plantarum OLL2712* has shown a favorable safety profile during long-term use, whereas some strains may contribute to microbial imbalance (77). Overall, many Lactobacillus-containing probiotic formulations have demonstrated favorable safety profiles, particularly for well-characterized strains. However, long-term safety evaluation should remain strain-specific and population-specific, especially in vulnerable individuals. Future clinical studies should include whole-genome sequencing to verify strain identity and screen for virulence factors, mobile antibiotic resistance genes, plasmids, and other transferable genetic elements. Phenotypic antibiotic susceptibility testing, standardized quality control of probiotic formulations, adverse-event monitoring, and long-term follow-up should also be incorporated before Lactobacillus-related interventions can be considered for routine clinical use in autoimmune thyroid diseases.

### 6.4 Incomplete mechanistic understanding: a barrier to precision application

The mechanisms by which specific Lactobacillus strains or Lactobacillus-containing probiotic formulations modulates AITDs remain incompletely understood, limiting its rational and targeted clinical application. Several key issues persist. First, the molecular pathways linking the gut–thyroid axis have not been clearly elucidated. Although some evidence suggests that certain Lactobacillus strains may influence thyroid-related immuneresponses, the strain-specific metabolic products and signaling pathways involved, including Toll-like receptor activation and SCFA-mediated mechanisms, remain unclear (58). Second, the mechanisms through which specific probiotic strains influence thyroid autoantibody production remain unresolved. While studies have shown that *Bifidobacterium longum* can reduce TRAb levels in GD patients (44), it is still unknown whether this occurs through modulation of B cell activation or alterations in T follicular helper cell differentiation. Additionally, discrepancies between *in vitro* and *in vivo* findings limit the generalizability of current evidence. For instance, while *in vitro* studies have shown that certain probiotic strains suppress autoreactive T cell proliferation, these effects have only been observed in animal models for a limited number of strains (58). Moreover, the influence of Lactobacillus on peripheral thyroid hormone metabolism is poorly characterized. One study noted potential involvement in regulating UDP-glucuronosyltransferase gene expression, but the upstream regulatory networks remain undefined (59). The lack of strain-specific mechanistic insight impedes the ability to tailor strain selection and dosing based on individual host characteristics, thereby restricting the broader application genomics, and immune–metabolic axes to shift probiotic therapy from empirical to mechanism-driven practice.

### 6.5 Discordant lactobacillus signatures and evidence heterogeneity in AITDs

A notable challenge in translating Lactobacillus-related findings into clinical strategies for autoimmune thyroid diseases is the inconsistent direction of Lactobacillus alterations across existing studies. Although several studies and pooled analyses have reported a reduced abundance of certain Lactobacillus strains in patients with autoimmune thyroid diseases, other cohorts, particularly those involving Graves’ disease, have observed increased certain Lactobacillus strains abundance (47, 48, 51). These findings should not be interpreted as a simple contradiction, but rather as evidence that Lactobacillus signatures are highly context-dependent.

Several sources of heterogeneity may explain these divergent observations. First, autoimmune thyroid diseases include biologically distinct entities, mainly Graves’ disease and Hashimoto’s thyroiditis, which differ in thyroid hormone status, immune activation patterns, intestinal motility, metabolic state, and treatment exposure (78, 79). Therefore, microbial changes observed in hyperthyroid Graves’ disease may not be directly comparable with those in hypothyroid or euthyroid Hashimoto’s thyroiditis (80). Second, treatment status is an important confounder. Antithyroid drugs, levothyroxine replacement, disease duration, and restoration of thyroid function may all reshape the intestinal ecosystem (81). Third, most available studies report relative abundance at the genus level. Lactobacillus comprises multiple species and strains with distinct immunomodulatory and metabolic properties; therefore, genus-level enrichment does not necessarily indicate a uniformly beneficial effect. Moreover, an apparent increase in relative abundance may reflect compositional shifts caused by depletion of other taxa rather than a true absolute expansion of Lactobacillus.

These considerations have important clinical implications. Observational enrichment of certain Lactobacillus strains in Graves’ disease should not be directly interpreted as evidence against probiotic supplementation, just as reduced certain Lactobacillus strains abundance in other AITD cohorts should not be viewed as sufficient justification for Lactobacillus-based therapy. Instead, current evidence suggests that Lactobacillus may act as a context-dependent microbial marker whose functional significance depends on disease subtype, thyroid functional status, host immune background, strain-level characteristics, and the surrounding microbial community (47). Future studies should therefore stratify patients by AITD subtype, thyroid hormone status, antibody profile, treatment exposure, diet, iodine intake, and recent antibiotic or probiotic use. Longitudinal designs integrating strain-resolved metagenomics, absolute quantification, metabolomics, and immune phenotyping are needed to determine whether Lactobacillus changes are causal contributors, compensatory responses, or secondary markers of thyroid-related dysbiosis.

### 6.6 Evidence gaps in disease-specific mechanistic validation

Another important challenge is that many proposed mechanisms linking Lactobacillus to autoimmune thyroid diseases are inferred from non-AITD experimental systems. Studies in colitis, obesity, Parkinson’s disease, and non-mammalian models have shown that certain Lactobacillus strains may influence intestinal barrier integrity, microbial metabolites, systemic inflammation, and Treg/Th17 immune balance (30, 33–35, 37). However, these models differ substantially from AITDs in disease-specific antigens, endocrine status, immune microenvironment, host metabolism, and tissue targets. Therefore, such findings provide biological plausibility but should not be considered direct evidence that the same mechanisms operate in Hashimoto’s thyroiditis or Graves’ disease.

At present, AITD-specific evidence remains limited. Human studies mainly demonstrate associations between altered gut microbiota and clinical or immunological features of AITDs, while causal relationships remain insufficiently established. A small number of animal studies, particularly those using TSH receptor-induced Graves’ disease or Graves’ orbitopathy models, provide more disease-relevant evidence that microbiota modulation can influence thyroid autoimmunity-related phenotypes (47, 48, 51). Nevertheless, these findings still require validation in independent models and human interventional studies. Future mechanistic studies should prioritize AITD-specific designs, including longitudinal patient cohorts, strain-resolved metagenomics, metabolomics, immune phenotyping, intestinal barrier assessment, and intervention studies stratified by disease subtype, thyroid hormone status, antibody profile, and treatment exposure.

## 7 Future research priorities and translational considerations for lactobacillus-related interventions in AITDs

Current clinical evidence supporting Lactobacillus-related interventions in AITDs remains preliminary. Existing studies are limited by small sample sizes, short follow-up periods, heterogeneous probiotic formulations, variable strain composition, inconsistent dosing regimens, and differences in background thyroid treatment. Therefore, the available evidence is insufficient to recommend Lactobacillus therapy as part of routine AITD management. The following perspectives should be interpreted as research priorities and translational considerations rather than clinical recommendations.

### 7.1 Toward personalized probiotic research: from general supplementation to strain-specific evaluation

Individual variability in response to specific Lactobacillus strains and probiotic formulations represents a major challenge for future clinical evaluation in AITDs. This heterogeneity may arise from multiple factors, including strain-specific functional properties, baseline gut microbiota composition, host genetic background, comorbid conditions, thyroid disease subtype, concomitant medication, and differences in intervention strategies. Therefore, future studies should move beyond general probiotic supplementation and evaluate strain-specific effects using standardized formulations and predefined clinical and immunological endpoints. For example, *Lactiplantibacillus plantarum OLL2712* cultured at pH 6.5 and 37 °C has been shown to enhance IL-10 expression, suggesting that culture conditions may influence the immunomodulatory activity of specific strains (77). Similarly, response surface methodology has been used to optimize the fermentation parameters of *Limosilactobacillus fermentum CECT5716* and enhance its acid-producing capacity and probiotic activity (82). These findings indicate that strain preparation and culture conditions may affect biological function. However, whether such optimized strains improve thyroid autoimmunity, thyroid function, or clinical outcomes in AITD patients remains unknown.

Innovative delivery systems may also improve strain viability in the gastrointestinal environment. Microencapsulation using dual-layer coating technology has increased *Lactococcus lactis* survival rates to over 85% in simulated gastric fluid, compared with less than 20% for non-encapsulated strains (83). In addition, 3D-printed formulations have shown potential for improving colonic delivery and intestinal persistence of selected probiotic strains (84). Importantly, formulation design should consider gut environmental differences in specific populations. For example, acid-resistant formulations may be particularly beneficial for hypothyroid patients, whose intestinal pH tends to be lower (58). These findings suggest that rational selection of strain-specific probiotic candidates and formulation strategies tailored to host-specific conditions will be critical for advancing clinical translation. Although these technologies may help standardize future probiotic interventions, their clinical relevance to AITDs requires direct validation in adequately powered trials.

### 7.2 Development of candidate functional strains: a multi-dimensional screening framework

The identification of safe and functionally characterized Lactobacillus strains is essential before disease-specific clinical testing can be considered. Current screening strategies evaluate strain-dependent characteristics, including immunomodulatory capacity, mucosal adhesion ability, metabolic activity, genomic safety, and biosafety profiles. Key approaches include high-throughput functional screening, whole-genome sequencing, metabolomic profiling, and validation of strain-specific biological activity. For example, *Limosilactobacillus fermentum CECT5716*, isolated from the feces of healthy infants, promotes IL-10 secretion while suppressing TNF-α production, suggesting immunoregulatory activity of this specific strain (85). *Lactiplantibacillus plantarum MH-301*, derived from traditional fermented foods, exhibits antimicrobial activity against Propionibacterium acnes and strain-specific immunomodulatory properties (86).

Genomic tools have further improved strain characterization. The complete genome of *Lactiplantibacillus plantarum IBB3423* revealed multiple adhesion-related genes, suggesting a potential contribution to intestinal adhesion capacity (87). Meanwhile, Beyond Lactobacillus, other lactic acid bacteria such as *Lactococcus lactis NIAIC59*, screened from yogurt using a combination of 16S rRNA sequencing and metabolomics, shows significant plasminogen-activating activity (88). However, these findings are not AITD-specific and should not be interpreted as evidence of clinical efficacy in thyroid autoimmunity. Future strain selection for AITD-related research should include disease-relevant endpoints, such as thyroid autoantibodies, thyroid hormone profiles, inflammatory markers, Treg/Th17 balance, intestinal barrier markers, and safety outcomes. For strains from non-traditional sources, such as aquatic environments, rigorous toxicological evaluation is essential to exclude pathogenic or transferable resistance risks (89). In summary, integrating strain-specific functional screening, multi-omics characterization, and rigorous safety assessments provides a promising framework for identifying candidate probiotic strains with potential relevance to AITD-associated immune modulation.

### 7.3 Combination with conventional AITD treatments: an investigational adjunctive strategy

The combination of selected probiotic strains with conventional thyroid treatments remains an investigational area. Preliminary studies suggest that certain probiotic formulations may influence immune markers, thyroid function, quality-of-life parameters, or gut microbiota composition when used alongside standard therapy. However, these findings are inconsistent and should not be considered sufficient to support routine adjunctive probiotic use in AITDs. For example, one study using a specific *Bifidobacterium longum* strain reported that co-administration with methimazole was associated with reduced TRAb levels in patients with Graves’ disease, with greater TRAb reduction than methimazole monotherapy in that study (44). Similarly, supplementation with selected probiotic formulations combined with levothyroxine has been reported to influence quality-of-life parameters and dose adjustment requirements in some patients with hypothyroidism (62). In pregnant women with hypothyroidism and coexisting small intestinal bacterial overgrowth, a combined intervention involving selected probiotics, prebiotics, and levothyroxine was associated with reduced TSH levels and changes in gut microbial composition (63). Despite these observations, the available studies are limited by small sample sizes, heterogeneous interventions, short follow-up durations, and variable probiotic compositions. Individual responses also differ substantially, with some patients showing apparent benefit and others showing minimal change (83). Therefore, future studies should evaluate probiotic adjuncts using standardized strain identification, fixed dosing regimens, appropriate control groups, longer follow-up, and clinically meaningful AITD-specific outcomes.

### 7.4 Integration with immunomodulatory strategies: a preclinical and exploratory research direction

The integration of selected probiotic strains with immunomodulatory strategies represents a theoretical and exploratory research direction rather than an established therapeutic approach for AITDs. Proposed mechanisms may involve strain-dependent immune regulation, modulation of the gut immune microenvironment, and microbiota–immune interactions. However, these concepts remain largely extrapolated from preclinical studies or non-AITD disease contexts. Studies have shown that specific *Bifidobacterium longum* strains may enhance the efficacy of PD-1 inhibitors in thyroid cancer by modulating gut microbiota and suppressing inflammatory pathways (90). In addition, certain Lactobacillus strains have been investigated as potential adjuvants for cancer vaccines in preclinical models (91). In experimental AITD models, administration of specific Lactobacillus strains combined with interleukin-2 has been reported to increase regulatory T cell populations, suggesting a possible role in immune tolerance restoration (92). Certain immunomodulatory approaches targeting CTLA-4-related pathways have been explored in combination strategies, although their effects on TRAb levels require further validation (44). However, in immunocompromised individuals, probiotic-based immunotherapy may pose infection risks, requiring careful assessment (71). Therefore, any future investigation of probiotic–immunomodulatory combinations should include strict safety monitoring, immune phenotyping, microbiota profiling, and strain-level characterization.

### 7.5 Clinical translation and standardization: requirements before routine use

The clinical translation of Lactobacillus-related interventions in AITDs faces several unresolved challenges. These include discrepancies between *in vitro* efficacy and *in vivo* performance, strain-dependent survival and colonization capacity, individualized dosing requirements, host compatibility, product quality control, and variability in probiotic formulations (58). At present, the lack of standardized probiotic strains, doses, treatment duration, and outcome measures limits comparability across studies. Emerging technologies, including multi-omics analysis, artificial intelligence-assisted strain selection, and microbiome engineering, may help identify candidate strains and clarify host–microbiota interactions (93). Gene editing and synthetic biology may also enhance specific bacterial functions, although these approaches raise ethical and biosafety concerns (82). However, these technological advances should be viewed as tools for future research rather than evidence supporting current clinical implementation.

Future development in this field will require multidisciplinary collaboration, robust ethical oversight, and standardized clinical evaluation systems incorporating strain identification, genomic characterization, safety assessment, and dose–response evaluation. The synergistic application of selected probiotic strains with immunotherapy may expand therapeutic possibilities for AITDs and contribute to the development of next-generation biotherapeutic platforms.
